# Selenium (Se) and breast cancer risk

**DOI:** 10.1186/1897-4287-10-S4-A4

**Published:** 2012-12-10

**Authors:** Katarzyna Jaworska - Bieniek, Anna Jakubowska, Katarzyna Durda, Tomasz Huzarski, Pablo Serrano-Fernandez, Grzegorz Sukiennicki, Magdalena Muszyńska, Tomasz Byrski, Jacek Gronwald, Satish Gupta, Katarzyna Kaczmarek, Jan Lubiński

**Affiliations:** 1 Pomeranian Medical University and Read Gene SA, Szczecin, Poland

## Aim of the study

The aim of the study was determination of serum Se concentration and identification of genetic variations in genes related to metabolism of selenium as markers of cancer risks for carriers of BRCA1 gene mutation and individuals with susceptibility to unselected breast cancer.

## Material and methods

Eight genotypes of 4 most common SNPs localised in GPX1, GPX4, TXNRD2 and SEP15 were selected. Genotyping was performed in 27 triplets matched BRCA1 carriers (cases and controls were matched 1:2) as well as on pairs matched 1:1 consisting of 220 unselected breast cancer (BRCA1 excluded). Cases and controls were matched for year of birth (+/-3 years), number and location of cancer among Iº relatives , smoking - the number of pack years (+/- 10%) and adnexectomy.

For group of 27 triplets matched BRCA1 carriers, all patients were carriers of one of three Polish founder BRCA1 mutation (C61G, 4153delA, 5382insC) and serum was collected 1 – 2 years before tumor diagnosis. Whereas for 220 pairs of unselected breast cancer, serum was collected before treatment but during diagnosis.

The following techniques for laboratory analyses have been applied: a) sequencing on ABI310, b) TaqMan analysis (a melting-curve genotyping with fluorescence-labeled probes based on the LightCycler 480 System (Roche Applied Science), c) determination of selenium concentration in serum using ICP MS – inductively coupled plasma mass spectrometry with ± 5% accuracy (Perkin Elmer).

## Results

In both studied groups there was observed that the optimal concentration of selenium was in range 100 – 120µg/l of serum independently on variants in selenoprotein coding genes.

If selenium level data were combined with some selenoprotein genotypes a tendency was found that the optimal selenium level for carriers of GPX1 nCC genotype (Table.1) is around 75µg/l of serum and for carriers of GPX1 CC the optimal selenium level is 120µg/l.

**Table 1:** Correlation between breast cancer risk and serum selenium concentration in carriers of GPX1 nCC genotype:

BRCA1 gene mutation carriers				
**Cancer**	**Quartiles**	**Concentration Se (µg/l)**	**No.**	**No.**
	
			**cases**	**controls**

**Breast (BRCA1)**	**I**	**52,37 – 70,45**	**4**	**4**
	
	II	72,45 – 78,03	1	7
	
	**III**	**78,68 – 85,5**	**2**	**6**
	
	**IV**	**88,5– 741,33**	**3**	**5**

Unselected breast cancer				

**Cancer**	**Quartiles**	**Concentration Se (µg/l)**	**No.**	**No.**
	
			**cases**	**controls**

**Unselected breast cancer**	**I**	**49,73 – 72,51**	**29**	**26**
	
	II	72,63 – 82,73	20	36
	
	**III**	**83,00 – 95,76**	**27**	**26**
	
	**IV**	**96,74 – 160,97**	**26**	**30**

## Conclusions

• Selenium, depending on Se concentration, can cause cancer or prevent against cancer.

• The effect of selenium depends on selenoprotein genotypes and concentration of selenium in the body/diet.

• Generally the optimal concentration of selenium for BRCA1 mutation carriers and unselected breast cancer is in range 100 – 120µg/l of serum.

• For women (BRCA1 mutation carriers and unselected breast cancer) with genotype nCC in GPX1 gene the optimal concentration of selenium is around 75µg/l of serum.

• The results of association studies require confirmation by a prospective observation of large groups of patients.

• Investigation on larger number of patients are needed especially valuable may be observations of women with increased risk of cancers.

